# Clinical and molecular characteristics of patients with brain metastasis secondary to pancreatic ductal adenocarcinoma

**DOI:** 10.1093/oncolo/oyae182

**Published:** 2024-07-16

**Authors:** Mahmoud Yousef, Mark W Hurd, Abdelrahman Yousef, Ethan B Ludmir, Ashwathy B Pillai, Jennifer Peterson, Eugene J Koay, Sali Albarouki, Ching-Wei Tzeng, Rebecca Snyder, Matthew H G Katz, Huamin Wang, Michael J Overman, Anirban Maitra, Shubham Pant, Brandon G Smaglo, Robert A Wolff, James Yao, John P Shen, Dan Zhao

**Affiliations:** Department of Gastrointestinal Medical Oncology, The University of Texas MD Anderson Cancer Center, Houston, TX, United States; Sheikh Ahmed Center for Pancreatic Cancer Research, The University of Texas MD Anderson Cancer Center, Houston, TX, United States; Department of Gastrointestinal Medical Oncology, The University of Texas MD Anderson Cancer Center, Houston, TX, United States; Department of Gastrointestinal Radiation Oncology, The University of Texas MD Anderson Cancer Center, Houston, TX, United States; Department of Hospital Medicine, The University of Texas MD Anderson Cancer Center, Houston, TX, United States; Department of Gastrointestinal Medical Oncology, The University of Texas MD Anderson Cancer Center, Houston, TX, United States; Department of Gastrointestinal Radiation Oncology, The University of Texas MD Anderson Cancer Center, Houston, TX, United States; Department of Gastroenterology and Hepatology, Baylor College of Medicine, Houston, TX, United States; Department of Surgical Oncology, The University of Texas MD Anderson Cancer Center, Houston, TX, United States; Department of Surgical Oncology, The University of Texas MD Anderson Cancer Center, Houston, TX, United States; Department of Surgical Oncology, The University of Texas MD Anderson Cancer Center, Houston, TX, United States; Department of Anatomical Pathology, The University of Texas MD Anderson Cancer Center, Houston, TX, United States; Department of Gastrointestinal Medical Oncology, The University of Texas MD Anderson Cancer Center, Houston, TX, United States; Department of Anatomical Pathology, The University of Texas MD Anderson Cancer Center, Houston, TX, United States; Department of Gastrointestinal Medical Oncology, The University of Texas MD Anderson Cancer Center, Houston, TX, United States; Department of Gastrointestinal Medical Oncology, The University of Texas MD Anderson Cancer Center, Houston, TX, United States; Department of Gastrointestinal Medical Oncology, The University of Texas MD Anderson Cancer Center, Houston, TX, United States; Department of Gastrointestinal Medical Oncology, The University of Texas MD Anderson Cancer Center, Houston, TX, United States; Department of Gastrointestinal Medical Oncology, The University of Texas MD Anderson Cancer Center, Houston, TX, United States; Department of Gastrointestinal Medical Oncology, The University of Texas MD Anderson Cancer Center, Houston, TX, United States

**Keywords:** pancreatic cancer, brain metastases, KRAS, PDAC, genetic testing, mutation

## Abstract

**Background:**

The prognosis for patients with pancreatic ductal adenocarcinoma (PDAC) is poor. Secondary brain metastasis (Br-M) occurs in less than 1% of patients. Clinical characteristics and molecular alterations have not been characterized in this rare patients’ subset.

**Materials and methods:**

The Foundry software platform was used to retrospectively query electronic health records for patients with Br-M secondary to PDAC from 2005 to 2023; clinical, molecular, and overall survival (OS) data were analyzed.

**Results:**

Br-M was diagnosed in 44 patients with PDAC. Median follow-up was 78 months; median OS from initial PDAC diagnosis was 47 months. Median duration from PDAC diagnosis to Br-M detection was 24 months; median OS from Br-M diagnosis was 3 months. At Br-M diagnosis, 82% (*n* = 36) of patients had elevated CA19-9. Lung was the most common preexisting metastatic location (71%) with Br-M, followed by liver (66%). Br-M were most frequently observed in the frontal lobe (34%, *n* = 15), cerebellar region (23%, *n* = 10), and leptomeninges (18%, *n* = 8). *KRAS* mutations were detected in 94.1% (*n* = 16) of patients who had molecular data available (*n* = 17) with KRAS^G12V^ being the most frequent subtype 47% (*n* = 8); KRAS^G12D^ in 29% (*n* = 5); KRAS^G12R^ in 18% (*n* = 3). Patients who underwent Br-M surgical resection (*n* = 5) had median OS of 8.6 months, while median OS following stereotactic radiosurgery only (*n* = 11) or whole-brain radiation only (*n* = 20) was 3.3 and 2.8 months, respectively.

**Conclusion:**

Br-M is a late PDAC complication, resulting in an extremely poor prognosis especially in leptomeningeal disease. *KRAS* was mutated in 94.1% of the patients and the KRAS^G12V^ subtype was prevalent.

## Teaser

Brain metastasis secondary to pancreatic adenocarcinoma is rare. This article describes the clinical characteristics of a relatively large cohort of patients and provides information on the disease course, molecular features, treatment patterns, and prognosis of these patients.

**Implications for Practice**

Brain metastasis secondary to pancreatic adenocarcinoma is rare, and clinical and molecular characterization of patients with this diagnosis is limited. Here, the clinical characteristics of a relatively large cohort of patients (*n* = 44), including molecular characteristics in a subset (*n* = 17), were identified, and reported. Data presented in this study provide information on the disease course, molecular features, treatment patterns, and prognosis of these patients, which could inform potential treatment approaches and future development of new therapies for brain metastatic patients, including KRAS-targeting systemic therapies that have higher ability to cross the blood-brain barrier.

## Introduction

Pancreatic ductal adenocarcinoma (PDAC) remains one of the most difficult cancers to treat as it is often discovered at late stages.^1-3^ Prognosis is generally poor with a 1-year survival of less than 20% overall and a 5-year survival rate of less than 5% for metastatic disease.^2^ Current predictions suggest that the incidence of PDAC will become more prevalent, with associated mortality second only to lung cancer.^2,4^ Treatment for PDAC typically involves standard chemotherapy and potentially radiation; for those fortunate enough to be identified early in the disease progression, chemotherapy and surgical resection have improved survival.^2^

Liver, lung, lymph nodes, and peritoneum are the most common locations of metastasis.^5^ Hematogenous spread of PDAC to other locations such as bone, kidneys, and brain has been reported, but currently, published data characterizing patients with brain metastasis (Br-M) from PDAC are limited.^6^ The pattern of PDAC metastasis is associated with disease outcomes.^6^ Br-M secondary to PDAC is relatively rare and happens in less than 1% of patients.^7,8^ Previous reports indicate an even worse survival in patients with PDAC who developed Br-M,^9^ but limited clinical and molecular data are available.

Effective treatment for PDAC is urgently needed. *KRAS* is mutated in nearly all PDAC patients, and more than 90% of these *KRAS* mutations are at codon 12, including 35% *KRAS*^G12D^, 30% *KRAS*^G12V^, 15% *KRAS*^G12R^, and 1%-2% *KRAS*^G12C^.^10-12^ Targeting KRAS had been challenging until the discovery of allosteric KRAS^G12C^ mutant-specific inhibition by covalent binding to the mutant cysteine beneath the switch-II region, which locks it in the inactive GDP bound form.^13^ KRAS^G12C^ inhibitors sotorasib (AMG510) and adagrasib (MRTX849) were approved by the FDA for use in lung cancer, and efficacy of these drugs in pancreatic cancer has been reported.^14-17^ Moreover, pan-KRAS inhibitor RMC-6236 (NCT05379985) and *KRAS*^*G12D*^ inhibitor MRTX1133 also showed promising results.^18^

For patients with Br-M secondary to PDAC, penetration of the blood-brain barrier is a challenge for systemic treatment approaches. However, little is known about the molecular features of patients with PDAC with Br-M, particularly regarding KRAS mutations; characterization of KRAS mutations in this patient population could indicate a role for KRAS-targeted therapy in the future. Recent advances in next generation sequencing (NGS) and the update of mutation testing in clinical practice now provide more information on molecular features. With the development of data science and a software platform for integrated extraction and analysis of clinical data, molecular testing/NGS results, as well as tumor registry data, we were able to identify a cohort of patients with the relatively uncommon Br-M from PDAC from a large database.^19,20^ Here we present our institutional experience and the clinical and genomic characterization of patients with Br-M secondary to PDAC.

## Materials and methods

### Study population

A retrospective analysis was performed on patients with histologically confirmed PDAC seen at MD Anderson from January 1, 2005 to January 1, 2023, using the Foundry software platform (Palantir Technologies, Denver, CO)^19-21^ to identify patients with Br-M. This study was approved by the University of Texas MD Anderson Cancer Center Institutional Review Board (IRB) under protocol number 2023-0091. Informed consent was waived, as per the IRB guidelines for retrospective studies of previously collected clinical and molecular information.

### Clinical data and molecular profiling

Patient demographics, clinical characteristics, available molecular data, and surgical history were extracted from the electronic health record (EHR). Follow-up data were collected from the date of diagnosis until June 2023. Disease stage at the time of diagnosis was collected from clinic notes and chart review. Vital status was determined from the EHR and administrative death indices, with overall survival (OS) calculated from both date of primary diagnosis and date of Br-M until death or last contact.

Patients with concomitant metastatic disease from cancers other than PDAC were excluded. Performance status was collected from the EHR at the time of Br-M diagnosis. The last CA19-9 measurement within 6 months prior to the diagnosis of Br-M was included for analysis. Sites of Br-M, leptomeningeal involvement, and the number of brain lesions at diagnosis were extracted from brain imaging at the time of Br-M diagnosis.

Molecular testing had been performed at MD Anderson’s molecular diagnostics laboratory,^22^ which is College of American Pathologists accredited and Clinical Laboratory Improvement Amendments certified,^22,23^ for a subset of the identified patients. Germline variants were identified using matched tumor-normal sequencing. A 5-tier classification system was applied to all genetic alterations with only variants classified as pathogenic or likely pathogenic retained.^24^ The dataset was deidentified prior to analysis.

### Statistical analysis

Baseline characteristics of the study population were summarized. Time-to-event endpoints were visualized using Kaplan-Meier curves, and differences in OS were compared using the log-rank test. Univariate Cox regression models were fit to analyze the hazard ratios comparing all-cause mortality for different brain-directed therapies. All statistical analyses were performed in GraphPad Prism, version 9.0 (GraphPad Software, San Diego, CA), R Studio Version 3.1.0 (RStudio, PBC, Boston, MA) and the Palantir Foundry platform (Palantir Technologies, Denver, CO). All tests were 2-sided, and statistical significance was identified by a *P*-value < .05.

## Results

### Patient characteristics

A total of 44 patients with PDAC and secondary Br-M were identified, and median follow-up time was 78 months. The median age at PDAC diagnosis was 61.5 years. The patients were predominantly male (61%; *n* = 27) with most being non-Hispanic White (84%, *n* = 37) or Hispanic patients (9%, *n* = 4); 14% (*n* = 6) had a history of other localized cancer diagnoses. Median OS from initial PDAC diagnosis was 47 months (95% CI = 34-79 months). Nearly half of the patients (48%, *n* = 21) had metastatic disease at diagnosis while the others (52%, *n* = 23) initially had localized disease (Table 1). Median OS for patients with metastatic disease was 20 months (95% CI = 6.4-NA months), while for patients with initial diagnosis of localized disease was 58 months (95% CI = 46.3-115.4 months). Median interval of Br-M diagnosis after initial diagnosis of metastatic disease was 9.2 months (IQR = 1.2-17.3 months), and median interval of 44.2 months (IQR = 30-62.6 months) was observed from initially diagnosis of localized disease to Br-M. However, median OS from Br-M diagnosis was similar for patients who had metastatic disease and localized disease at initial diagnosis, 3.3 and 2.9 months, respectively.

**Table 1.** Clinical characteristics (*n* = 44).

Characteristics	Number (%)
Age at primary diagnosis	
Mean (SD)	60.5 (9.67)
Median [min, max]	61.5 [29.0, 80.0]
Sex	
Female	17 (39%)
Male	27 (61%)
Race	
White or Caucasian	37 (84%)
Hispanic or Latino	4 (9%)
Asian	1 (2%)
Black or African American	1 (2%)
Unknown	1 (2%)
Other localized cancer diagnosis	
Yes	6 (14%)
No	38 (86%)
ECOG PS at Br-M	
0	7 (16%)
1	25 (57%)
2	8 (18%)
3	4 (9%)
CA_19-9 at Br-M	
Elevated	36 (82%)
Normal	8 (18%)
Stage at initial diagnosis	
1	6 (14%)
2	5 (11%)
3	12 (27%)
4	21 (48%)
Surgery for primary tumor	
Yes	19 (43%)
No	25 (57%)
Histological differentiation	
Poorly differentiated	19 (43%)
Moderate differentiated	17 (39%)
Well differentiated	1 (2%)
Unknown	7 (16%)
Follow-up from initial diagnosis	
Median FU (months)	78
95% CI	(46—NA)
Overall survival from initial diagnosis	
Median OS (months)	47
95% CI	(34-79)
Time from PDAC Diagnosis to Br-M	
Mean (SD) months	33.7 (30.2)
Median [min, max] months	24.1 [0, 141]
Overall survival from Br-M	
Median OS (months)	3.0
95% CI	(2-6)
Lines of therapy	
Median [min, max]	4.00 [0, 8.00]
Brain lobes involved	
Multiple	27 (61%)
Frontal	5 (11%)
Parietal	4 (9%)
Cerebellum	3 (7%)
Temporal	2 (5%)
Other	3 (7%)
Number of brain lesions	
1	14 (32%)
2	4 (9%)
3	3 (7%)
4	1 (2%)
5	2 (4%)
> 5	20 (46%)
Leptomeningeal involvement	
Yes	8 (18%)
No	36 (82%)
Symptoms of Br-M	
Headache	13 (30%)
Focal neurological deficit^a^	8 (19%)
AMS or confusion	7 (16%)
Trial screening (asymptomatic)	5 (12%)
General weakness or fatigue	4 (9%)
Seizure	3 (7%)
Other	3 (7%)
Metastasis to liver	
Yes	29 (66%)
No	15 (44%)
Metastasis to lung	
Yes	31 (70%)
No	13 (30%)
Metastasis to bone	
Yes	17 (39%)
No	27 (61%)
Metastasis to other sites	
Yes	13 (30%)
No	31 (70%)
Brain-directed therapy	
Yes	36 (82%)
No	8 (18%)
Type of brain-directed therapy	
Stereotactic radiosurgery	11 (25%)
Surgical resection	5 (11%)
WBRT	20 (46%)
None	8 (18%)

^a^Focal neurological deficit includes unilateral weakness (*n* = 1), unilateral paresthesia (*n* = 1), dysarthria (*n* = 3), or ataxia (*n* = 3).

Abbreviations: Br-M, brain metastasis; ECOG PS, Eastern Cooperative Oncology Group Performance Status; CA 19-9, Carbohydrate antigen 19-9; FU, Follow-up; PDAC, Pancreatic ductal adenocarcinoma.

### Secondary Br-M characteristics

**Figure 1.**
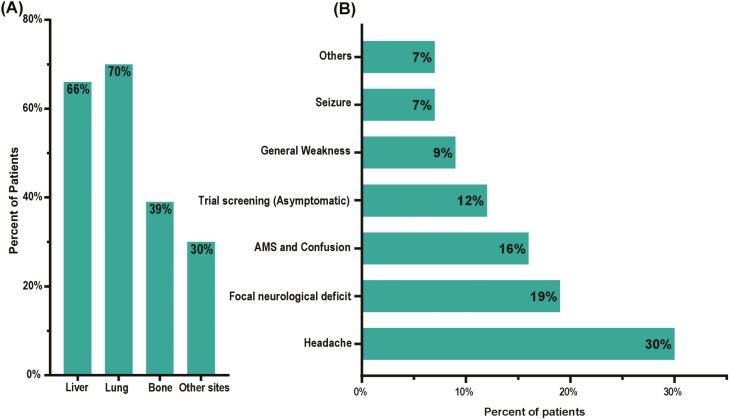
Patient clinical characteristics: (A) extracranial metastatic sites, (B) presenting symptoms for brain metastasis.

While the median duration for Br-M detection from the initial PDAC diagnosis was 24.1 months, (inter-quantile range [IQR] = 9.2-44.4 months), the median OS from Br-M diagnosis was only 3 months (95% CI = 2-6 months; Table 1). Resection of primary tumor was performed in 43% (*n* = 19) of patients. Median interval for the development of Br-M from the primary surgical resection was 42.9 months (IQR = 35.6-60.2 months) and 25.6 months (IQR = 6.9-41.9 months) from relapse after surgery to Br-M. All patients had at least one extracranial site of metastasis at the time of Br-M diagnosis. Seventy-three percent (*n* = 32) of patients had more than one extracranial metastatic site. Lung metastasis was the most common (70%, *n* = 31) followed by liver (66%, *n* = 29) and bone metastasis (39%, *n* = 17, Figure 1A). Histologic grade of the primary tumor was assessed for 37 patients; among these patients 51% (*n* = 19) had poor differentiation, while 46% (*n* = 17) had moderate differentiation and only 1 patient had a well-differentiated tumor. In addition, the majority (73%, *n* = 32) had Eastern Cooperative Oncology Group (ECOG) performance 0 or 1 at Br-M diagnosis while 18% (*n* = 8) and 9% (*n* = 4) had ECOG 2 and 3, respectively. Elevated CA19-9 levels prior to Br-M were detected in 82% (*n* = 36) of patients. Most of the patients in this cohort were treated with multiple lines of systemic treatment prior to Br-M development (median number of lines of therapy = 4).

**Figure 2.**
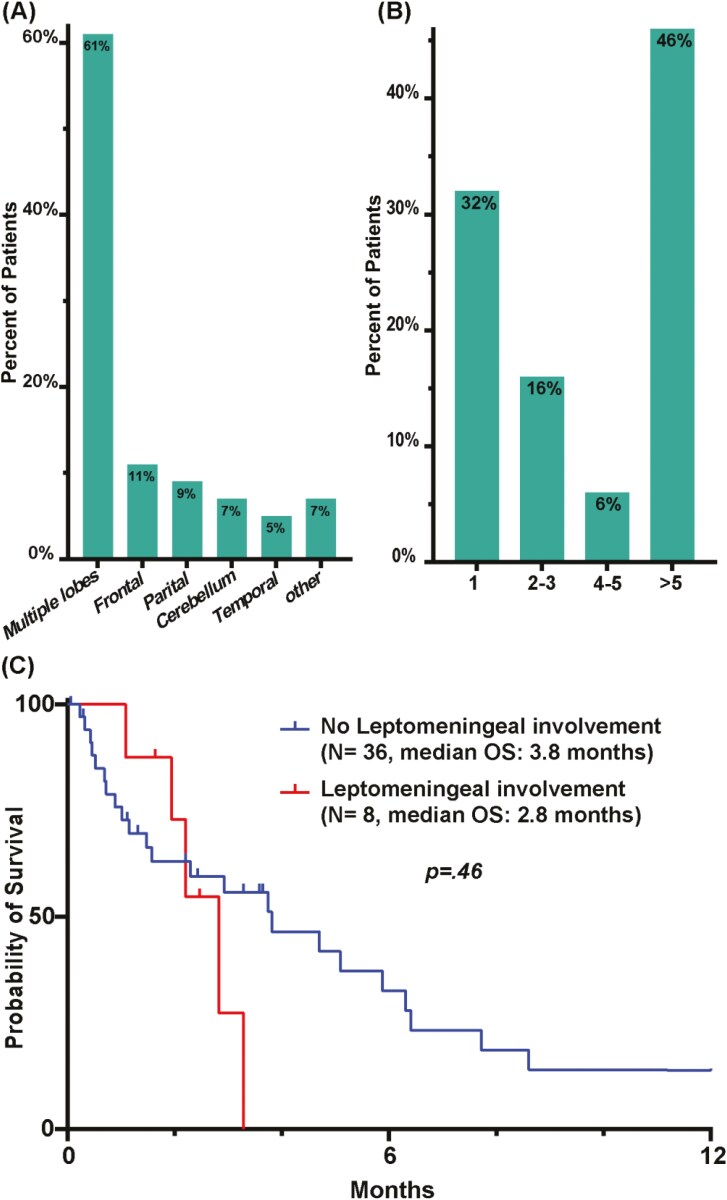
Brain metastases characteristics: (A) site of brain involvement at diagnosis, (B) number of brain lesions at diagnosis, (C) survival of patients with or without leptomeningeal disease.

The most common presenting Br-M symptoms were headache (30%, *n* = 13) followed by focal neurological deficits (19%, *n* = 8; Table 1). The neurological deficits included dysarthria (*n* = 3), ataxia (*n* = 3), unilateral weakness (*n* = 1), or unilateral paresthesia (*n* = 1), while altered mental status (AMS) was the presenting symptom for 16% (*n* = 7) of patients. A subset of patients (12%, *n* = 5) was diagnosed incidentally during clinical trial screening (Figure 1B). Most of the patients had multiple brain lobes involved at Br-M diagnosis (61%; *n* = 27). Frontal lobe-only involvement was detected in 11% (*n* = 5) of patients, followed by the parietal lobe (9%, *n* = 4), cerebellum (7%, *n* = 3) then temporal lobe (5%, *n* = 2, Figure 2A) involvement. Only 32% (*n* = 14) of patients had a single Br-M lesion at diagnosis. Unfortunately, 46% of the patients (*n* = 20) had more than 5 brain lesions at onset, limiting their available therapy options (Figure 2B). Additionally, leptomeningeal involvement was detected in 18% (*n* = 8) of patients at Br-M diagnosis. Compared to patients without leptomeningeal disease, patients with leptomeningeal disease showed a tendency toward poorer OS (median OS 2.8 months vs 3.8 months, HR = 1.35, 95% CI = 0.52-3.5, *P* = .46, Figure 2C) and no patient with leptomeningeal disease survived more than 4 months.

### Molecular profiling results

**Figure 3.**
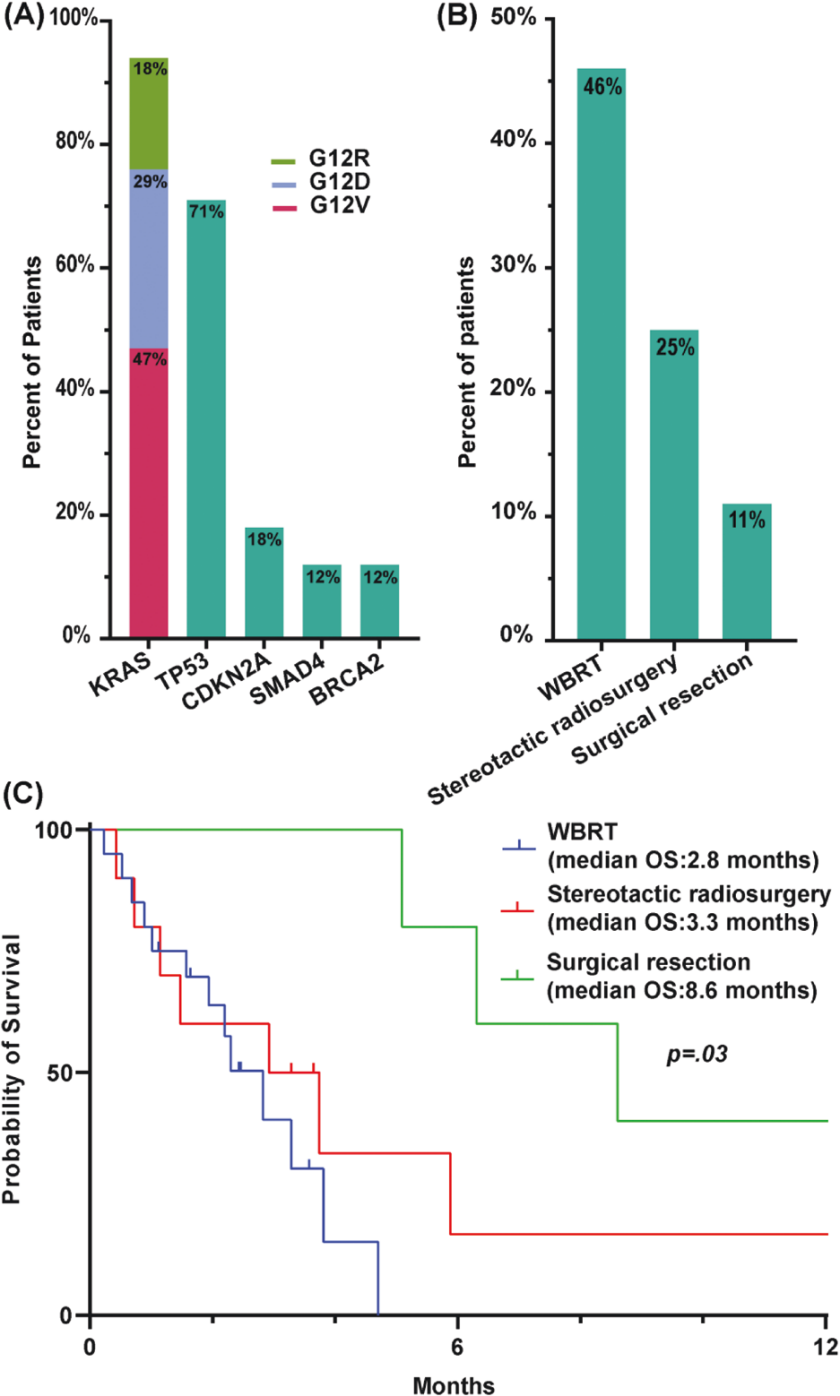
Molecular characteristics and brain-directed therapy: (A) mutation frequencies in patients who underwent clinical mutational testing, (B) types of brain metastasis-directed therapy, (C) overall survival stratified by types of brain-directed therapy.

Targeted clinical mutation testing was conducted in a subset of 17 patients. *KRAS* mutations were common (94.1%, *n* = 16). The most detected allele was KRAS^G12V^, which was found in 47% (*n* = 8) of the tested patients; KRAS^G12D^ was positive in 29% (*n* = 5), and KRAS^G12R^ in 18% (*n* = 3) of the patients tested. *TP53* was the second most prevalent mutation (71%, *n* = 12) followed by *CDKN2A* (18%, *n* = 3, Figure 3A). Other detected mutations included *SMAD4* (*n* = 2), *BRCA2* (*n* = 2), and *BRCA1* (*n* = 1, Table 2). The 3 patients with *BRCA* mutations all had germline *BRCA* mutations.

**Table 2.** Molecular profiling results (*n* = 17).

Gene	*n* (%)
KRAS	
Wildtype	1 (5.9%)
G12V	8 (47.1%)
G12D	5 (29.4%)
G12R	3 (17.6%)
TP53	
Wildtype	5 (29.4%)
Mutated	12 (70.6%)
CDKN2A	
Wildtype	14 (82.4%)
Mutated	3 (17.6%)
SMAD4	
Wildtype	15 (88.2%)
Mutated	2 (11.8%)
BRCA2	
Wildtype	15 (88.2%)
Mutated	2 (11.8%)
BRCA1	
Wildtype	16 (94.1%)
Mutated	1 (5.9%)
PDGFRA	
Wildtype	16 (94.1%)
Mutated	1 (5.9%)
ROS1	
Wildtype	16 (94.1%)
Mutated	1 (5.9%)
IDO2	
Wildtype	16 (94.1%)
Mutated	1 (5.9%)
STK11	
Wildtype	16 (94.1%)
Mutated	1 (5.9%)
PIK3CA	
Wildtype	16 (94.1%)
Mutated	1 (5.9%)
CHEK2	
Wildtype	16 (94.1%)
Mutated	1 (5.9%)
FBXW7	
Wildtype	16 (94.1%)
Mutated	1 (5.9%)
BAP1	
Wildtype	16 (94.1%)
Mutated	1 (5.9%)
CREBBP	
Wildtype	16 (94.1%)
Mutated	1 (5.9%)
NBN	
Wildtype	16 (94.1%)
Mutated	1 (5.9%)
CIC	
Wildtype	16 (94.1%)
Mutated	1 (5.9%)
CUX1	
Wildtype	16 (94.1%)
Mutated	1 (5.9%)
FGF6	
Wildtype	16 (94.1%)
Mutated	1 (5.9%)
LRP1B	
Wildtype	16 (94.1%)
Mutated	1 (5.9%)
SMC1A	
Wildtype	16 (94.1%)
Mutated	1 (5.9%)
NOTCH2	
Wildtype	16 (94.1%)
Mutated	1 (5.9%)
MLH3	
Wildtype	16 (94.1%)
Mutated	1 (5.9%)
CCNE1	
Wildtype	16 (94.1%)
Mutated	1 (5.9%)

### Brain-directed therapy for Br-M and associated outcomes

Thirty-six patients (82%) received treatment for Br-M. Eight patients did not receive any Br-M-related intervention. Whole-brain radiation therapy (WBRT) was the most commonly implemented intervention (*n* = 20, 46%) due to the presence of multiple brain lesions or the location of the lesions. The second most common intervention was stereotactic radiosurgery (*n* = 11, 25%).

Surgical resection was conducted on a small subset of patients (*n* = 5, 11%, Figure 3B). We summarized the clinical characteristics of these highly selected patients in Table 3. All patients had 1 or 2 brain lesions at Br-M diagnosis. The presenting signs for Br-M are listed; headache was observed in 4 out of 5 patients and AMS in 1 patient. This highly selected subset of patients who underwent surgical resection, also received either WBRT or stereotactic radiosurgery and their OS compared to other brain-targeted interventions was slightly better with a median of 8.6 months in the surgery cohort vs 3.3 months following stereotactic radiosurgery and 2.8 months for WBRT (overall log-rank *P* = .03, Figure 3C). Compared to WBRT, the subset of highly selected patients who were able to tolerate surgical resection had significantly better survival (HR = 0.17, 95% CI = 0.04-0.067, *P* = .01). Additionally, stereotactic radiosurgery compared to WBRT showed a tendency toward better survival (HR = 0.47, 95% CI = 0.16-1.30, *P* = .16).

**Table 3.** Characteristics of patients underwent surgical resection of the brain metastasis (*n* = 5).

Characteristics	*n*
Age at primary diagnosis	
Median [min, max]	57 [29, 65]
Other cancer diagnosis	
Yes^a^	1
No	4
ECOG at Br-M	
1	5
CA_19-9 at Br-M	
Elevated	3
Normal	2
Stage at initial diagnosis	
2	1
3	2
4	2
Surgery for primary tumor	
Yes	2
No	3
Histological differentiation	
Poorly differentiated	3
Moderate differentiated	2
Initial diagnosis to Br-M interval	
Median [min, max], months	39.7 [0, 70]
Lines of chemotherapy received	
Median (lines)	5
Brain lobes involved at diagnosis	
Multiple	1
Frontal	1
Parietal	1
Cerebellum	1
Temporal	1
Number of brain lesions at diagnosis	
1	2
2	3
Leptomeningeal involvement	
No	5
Symptoms related to Br-M	
Headache	4
AMS	1
Overall survival from initial diagnosis	
Median OS (months)	58
Overall survival from Br-M	
Median OS (months)	8.6

^a^One patient had history of resected peritoneal serous carcinoma with no evidence of disease.

## Discussion

The present study has presented, to the best of our knowledge, the largest series of patients with Br-M from PDAC to date, with clinical characteristics reported for 44 patients, and molecular information reported for 17 of the 44. While the median time from the initial PDAC diagnosis to Br-M was 24.1 months, median OS from the Br-M was detected was 3 months. The most common extracranial metastasis was lung metastasis (70%). Of those with molecular information available, the vast majority were positive for *KRAS* mutations (*n* = 16, 94.1%): 47% *KRAS*^G12V^ (*n* = 8), 29% *KRAS*^G12D^ (*n* = 5) and 18% *KRAS*^G12R^ (*n* = 3).

Our cohort of 44 patients with Br-M secondary to PDAC showed longer median OS from primary PDAC diagnosis of 47 months, and 1-year survival probability was 80.8% compared to only 30% 1-year survival probability for patients with PDAC reported previously.^25,26^ This difference should be weighed cautiously, given that the patients in our study were from a single tertiary cancer center with highly selected patient population, which could have resulted in an ascertainment bias. In addition, the duration from the diagnosis of the primary PDAC to the development of secondary Br-M was also relatively long, with median time of 25 months. These data suggested that Br-M is a late event of PDAC, usually occurring in patients with insidious disease progression following multiple lines of chemotherapy. Surgical resection of the primary tumor occurred in more than 50% of the patients, while usually there was less than 20% of patients were surgical candidates at initial diagnosis in pancreatic cancer. There is population bias and enrichment of surgical cases in this population, which is consistent with better OS in resected patients.^27^ The relatively long duration between the primary diagnosis and Br-M is consistent with previous literature, which was reported to be 17 and 29 months.^28,29^ Given the present data and published lierature, as well as improvements achieved in survival of patients with PDAC over time,^30^ the incidence of secondary Br-M is expected to increase over time. The use of brain MRI in clinical practice besides CT scan and screening brain MRI during clinical trials will no doubt enhance identification of these patients.

In clinical practice, brain imaging such as MRI is not routinely used for patients with asymptomatic PDAC. Due to concerns of possible risks of immune effector cell-associated neurotoxicity syndrome (ICANS) during recently developed promising cellular therapy programs in PDAC, such as anti-KRAS mutation TCR T cells and anti-Claudin 18.2,^31,32^ we adopted brain MRI in screening of asymptomatic patients for clinical trials using cellular therapy and immunotherapy which could cause ICANS. In this study, 5 patients (12%) were asymptomatic when diagnosed during clinical trial screening. The most common presentation of the Br-M in our cohort was headache in about one-third of the patients, and focal neurological deficits were observed in only 16% of the patients; these findings were consistent with previous reports.^28,33^ Along with the consistently poor prognosis accompanying the Br-M development, this indicates that the low incidence rate previously reported from national registries and case series, which was approximately 0.4%-0.6%,^34^ might be an underestimation of the true incidence, which was up to 7% in older autopsy studies.^35^

The majority of our patients had metastatic disease prior to the Br-M diagnosis with the lung being the most prevalent site of metastasis; these data differ from a previous report analyzing fewer patients.^28^ However, both series suggest a relatively higher prevalence of lung metastasis, which was 50% of patients in one report and 77% in our cohort. A national registry-based study reported an incidence of lung metastasis in 20% in patients with PDAC.^34^ Liver metastasis (more than 80%) is the most common metastatic site in PDAC associated with worse OS, while lung metastasis is relatively uncommon (15%-20%) and associated with longer OS.^34,36^ Thus, the prevalence of lung metastasis in the patients with Br-M is either due to an underlying difference in the biology of the primary tumor,^37^ epithelial plasticity regulation^38^ or the fact that patients with lung metastases tend to survive longer in comparison to patients with liver metastasis.^39^

Several actionable driver mutations have been associated with Br-M in multiple cancers including *EGFR*-mutated lung cancer, *HER2*-positive breast cancer and gastroesophageal adenocarcinoma, and *BRAF*-mutated melanoma.^40,41^ The development of targeted therapy and immunotherapy has improved outcomes; for example, in a series of Br-M from biliary tract cancers, *BRAF* mutation (30%) was associated with longer OS. BRAF mutation was observed in 2 patients with the longest OS from Br-M diagnosis (33.8 and 31.4 months) and 1 patient with a BRAF mutation received BRAF-targeted therapy (MEK inhibitor binimetinib and BRAF inhibitor encorafenib) had OS 99.8 months from primary diagnosis.^42^ Limited data were reported in PDAC, and a better understanding of genetic and tumor molecular testing data could guide treatment decisions.^27^ Mutational testing was conducted in a subset of 17 patients (40%) in our study. Almost all patients tested positive for *KRAS*^G12^ mutations (94.1%, 16/17). The *KRAS*^G12V^ (47.1%, 8/17) mutation was the most frequently reported, followed by *KRAS*^G12D^ (29.4%, 5/17) and *KRAS*^G12R^ (17.6%, 3/17) mutations. The prevalence of *KRAS* mutations is similar to that in previous reports of patients with PDAC.^10,43^ However, the frequency of *KRAS*^G12V^ in this cohort is numerically slightly higher than in previous PDAC cohorts where the *KRAS*^G12D^ mutation has been more prevalent.^19,44^ We previously reported worse OS in patients with *KRAS*^*G12D*^-mutated PDAC (median OS 20 months in the overall population); therefore, it is possible that a fraction of *KRAS*^*G12D*^-mutated patients did not survive long enough to develop Br-M (median time from diagnosis was 24.8 months in this study).^19^ Nevertheless, larger independent datasets are needed for the validation of our findings. Intracranial efficacy of *KRAS*^*G12C*^ inhibitors adagrasib and sotorasib has been reported in patients with Br-M secondary to lung cancer.^45,46^ Adagrasib had an objective response rate of 42%, disease control rate of 90%, progression-free survival of 5.4 months, and median OS of 11.4 months in patients with non–small cell lung cancer.^45^ With more KRAS-targeted therapies entering clinical development in PDAC, we are looking forward to seeing their activity in patients with PDAC with Br-M. To the best of our knowledge, our study is the largest report of KRAS mutation characterization in patients with Br-M secondary to PDAC, and these data could guide future clinical studies and practice. The ability to cross the blood-brain barrier might be considered an important aspect in the development of KRAS-targeted therapy in this population.

The frequency of other mutations in our cohort is similar to previous reports, with mutations observed in *TP53* (*70.6%*, *12/17*), *CDKN2A* (*17.6%*, *3/17*), and *SMAD4* (*11.8%*, *2/17*), of notice that compared to these reports, mutations associated with worse prognosis as SMAD4 and CDNK2A mutations^11,47,48^ were less prevalent in our cohort which suggested they may not survive long enough until the development of with Br-M. Interestingly, 17% of our patients tested positive for germline mutations with 5.9% *BRCA1* (*1/17*) and *BRCA2* (11.8%, 2/17). Previous reports in the literature found the germline mutation rate to be higher in patients with Br-M^28^; additionally, a case report found *BRCA2* to be frequently mutated in these patients.^49^ While it is possible that germline BRCA mutations might be associated with Br-M in other primary tumor sites such as the breast,^50^ it is also possible that this association is due to better survival of patients with germline *BRCA* mutations in PDAC.^51^ The brain-penetrant poly(ADP(adenosine diphosphate)-ribose) polymerase inhibitors (PARPi) and DNA damaging agents warrant more study in patients with Br-M in the future in BRCAs mutated patients.^52^

The prognosis after the development of Br-M was extremely poor, with median OS of 3 months in our cohort. Eight (18%) patients had leptomeningeal disease, with median OS of only 2.8 months, while the median OS was 3.8 months for patients without leptomeningeal disease. The extremely poor prognosis among patients with leptomeningeal disease is consistent with previous reports of 1.5-3 months OS.^28,33,53^ Unfortunately, treatment for leptomeningeal disease remains a challenge. In this study, only highly selected patients (*n* = 5, 11%) underwent surgery for oligometastatic disease, had relatively better OS (median OS 8.6 months). Multiple case reports have indicated better survival in patients who successfully underwent surgical resection of the Br-M.^29,41,54,55^ Given the improved prognosis of patients with oligometastatic disease, and the vague presentation of Br-M with headache as the most common presenting symptom, a high degree of suspicion is necessary to detect the metastasis early to improve the likelihood of surgical resection eligibility. While it may not be logistically feasible to screen all patients with PDAC for CNS involvement, our study provides guidance on the cases that should be selected for such screening and intervention to improve their outcome, such as patients with a relatively longer survival with PDAC, having multiple metastatic sites, especially lung involvement, and presenting with vague neurological symptom as persistent headache.

While this study represents a larger patient cohort compared to other reports in the literature, this sample of patients was also studied retrospectively and represents the experience of a single institution, which potentially impacts the generalizability of our findings. Further, only a subset of patients underwent genetic alterations testing in this study, which may limit our ability to detect statistical differences. Due to the limitations of the retrospective nature of our study, a prospective trial is needed to confirm the improved survival association observed in patients who underwent surgical resection. The patient variables that will enhance the treatment management approach need also additional attention. Nonetheless, despite the limitations of our study, we used a comparatively large cohort to gain insights that may aid physicians in the diagnosis and treatment of Br-M in patients with PDAC. Moreover, we identified genetic alterations that could guide the future development of targeted therapy in this population.

## Conclusion

This study reports the clinical and molecular features of a large cohort of patients with PDAC with Br-M; Br-M presented relatively late in the disease progression and was associated with extremely poor prognosis—particularly for those patients who had leptomeningeal disease. Concurrent lung metastasis was enriched in these patients. In this cohort, the frontal lobe was the most frequently affected central nervous system site. Additionally, *KRAS* was the most frequently detected mutation with the *KRAS*^G12V^ alteration being most commonly observed. Highly selected patients who underwent surgical resection for Br-M had relatively better OS.

## Data Availability

Individual patient-level data are not publicly available to maintain compliance with HIPPA regulations and IRB protocol. Anonymized data are available for noncommercial use from the corresponding author upon request pending data usage agreement and/or IRB-approved collaboration.

## References

[1] Ryan DP , Hong TS, Bardeesy N. Pancreatic adenocarcinoma. N Engl J Med. 2014;371(11):1039-1049. 10.1056/NEJMra1404198 25207767

[2] Sarantis P , Koustas E, Papadimitropoulou A, Papavassiliou AG, Karamouzis MV. Pancreatic ductal adenocarcinoma: treatment hurdles, tumor microenvironment and immunotherapy. World J Gastrointest Oncol. 2020;12(2):173-181. 10.4251/wjgo.v12.i2.173 32104548 PMC7031151

[3] Kamisawa T , Wood LD, Itoi T, Takaori K. Pancreatic cancer. Lancet. 2016;388(10039):73-85. 10.1016/S0140-6736(16)00141-0 26830752

[4] Huang J , Lok V, Ngai CH, et al. Worldwide burden of, risk factors for, and trends in pancreatic cancer. Gastroenterology. 2021;160(3):744-754. 10.1053/j.gastro.2020.10.007 33058868

[5] Peixoto RD , Speers C, McGahan CE, et al. Prognostic factors and sites of metastasis in unresectable locally advanced pancreatic cancer. Cancer Med. 2015;4(8):1171-1177. 10.1002/cam4.459 25891650 PMC4559028

[6] He C , Huang X, Zhang Y, Lin X, Li S. The impact of different metastatic patterns on survival in patients with pancreatic cancer. Pancreatology. 2021;21(3):556-563. 10.1016/j.pan.2021.01.014 33518454

[7] Go PH , Klaassen Z, Meadows MC, Chamberlain RS. Gastrointestinal cancer and brain metastasis: a rare and ominous sign. Cancer. 2011;117(16):3630-3640. 10.1002/cncr.25940 21319152

[8] Park K-S , Kim M, Park S-H, Lee K-W. Nervous system involvement by pancreatic cancer. J Neurooncol. 2003;63(3):313-316. 10.1023/a:1024337020884 12892239

[9] Lemke J , Scheele J, Kapapa T, et al. Brain metastasis in pancreatic cancer. Int J Mol Sci. 2013;14(2):4163-4173. 10.3390/ijms14024163 23429199 PMC3588092

[10] Waters AM , Der CJ. Kras: The critical driver and therapeutic target for pancreatic cancer. Cold Spring Harb Perspect Med. 2018;8(9):a031435. 10.1101/cshperspect.a031435 29229669 PMC5995645

[11] Cancer Genome Atlas Research Network. Integrated genomic characterization of pancreatic ductal adenocarcinoma. Cancer Cell. 2017;32(2):185-203 e113. 28810144 10.1016/j.ccell.2017.07.007 PMC5964983

[12] Bailey PChang DKNones K , et al. Genomic analyses identify molecular subtypes of pancreatic cancer. Nature. 2016;531(7592):47-52. 26909576 10.1038/nature16965

[13] Ostrem JM , Peters U, Sos ML, Wells JA, Shokat KM. K-Ras(g12c) inhibitors allosterically control GTP affinity and effector interactions. Nature. 2013;503(7477):548-551. 10.1038/nature12796 24256730 PMC4274051

[14] Canon J , Rex K, Saiki AY, et al. The clinical KRAS(g12c) inhibitor AMG 510 drives anti-tumour immunity. Nature. 2019;575(7781):217-223. 10.1038/s41586-019-1694-1 31666701

[15] Hallin J , Engstrom LD, Hargis L, et al. The KRAS^g12c^ inhibitor MRTX849 provides insight toward therapeutic susceptibility of KRAS-mutant cancers in mouse models and patients. Cancer Discov. 2020;10(1):54-71. 10.1158/2159-8290.CD-19-1167 31658955 PMC6954325

[16] Hong DS , Fakih MG, Strickler JH, et al. KRAS(g12c) inhibition with sotorasib in advanced solid tumors. N Engl J Med. 2020;383(13):1207-1217. 10.1056/NEJMoa1917239 32955176 PMC7571518

[17] Strickler JH , Satake H, George TJ, et al. Sotorasib in KRAS p.G12c-mutated advanced pancreatic cancer. N Engl J Med. 2022;388(1):33. 36546651 10.1056/NEJMoa2208470 PMC10506456

[18] Kemp SB , Cheng N, Markosyan N, et al. Efficacy of a small-molecule inhibitor of KrasG12d in immunocompetent models of pancreatic cancer. Cancer Discov. 2023;13(2):298-311. 10.1158/2159-8290.CD-22-1066 36472553 PMC9900321

[19] Yousef A , Yousef M, Chowdhury S, et al. Impact of KRAS mutations and co-mutations on clinical outcomes in pancreatic ductal adenocarcinoma. NPJ Precis Oncol. 2024;8(1):27. 38310130 10.1038/s41698-024-00505-0 PMC10838312

[20] Alfaro-Munoz K , Hallatt G, Sookprasong J, et al. Building a data foundation: How md anderson and palantir are partnering to accelerate research and improve patient care. J Clin Oncol. 2019;37(15_suppl):e18077-e18077. 10.1200/jco.2019.37.15_suppl.e18077

[21] Goldstein JB , Beird H, Zhang J, et al. Tackling “big data” for accelerating cancer research. J Clin Oncol. 2016;34(15_suppl):e23160-e23160. 10.1200/jco.2016.34.15_suppl.e23160

[22] Luthra R , Patel KP, Routbort MJ, et al. A targeted high-throughput next-generation sequencing panel for clinical screening of mutations, gene amplifications, and fusions in solid tumors. J Mol Diagn. 2017;19(2):255-264. 10.1016/j.jmoldx.2016.09.011 28017569

[23] Meric-Bernstam F , Brusco L, Shaw K, et al. Feasibility of large-scale genomic testing to facilitate enrollment onto genomically matched clinical trials. J Clin Oncol. 2015;33(25):2753-2762. 10.1200/JCO.2014.60.4165 26014291 PMC4550690

[24] Richards S , Aziz N, Bale S, et al.; ACMG Laboratory Quality Assurance Committee. Standards and guidelines for the interpretation of sequence variants: a joint consensus recommendation of the American College of Medical Genetics and Genomics and the Association for Molecular Pathology. Genet Med. 2015;17(5):405-424. 10.1038/gim.2015.30 25741868 PMC4544753

[25] Surveillance Research Program NCI. Data source(s): SEER incidence data, November 2022 submission (1975-2020), SEER 22 registries (excluding illinois and massachusetts). April 19, 2023. Updated November 16, 2023; cited March 25, 2024. https://seer.cancer.gov/statistics-network/explorer/

[26] Pishvaian MJ , Blais EM, Brody JR, et al. Overall survival in patients with pancreatic cancer receiving matched therapies following molecular profiling: a retrospective analysis of the know your tumor registry trial. Lancet Oncol. 2020;21(4):508-518. 10.1016/S1470-2045(20)30074-7 32135080 PMC7453743

[27] Park W , Chawla A, O’Reilly EM. Pancreatic cancer: a review. JAMA. 2021;326(9):851-862. 10.1001/jama.2021.13027 34547082 PMC9363152

[28] Emmet JJ , Maeve AL, Olca B, et al. Brain metastases in pancreatic ductal adenocarcinoma: assessment of molecular genotype–phenotype features—an entity with an increasing incidence? Clin Colorectal Cancer. 2018;17(2):e315-e321. 29496399 10.1016/j.clcc.2018.01.009 PMC6759921

[29] Kumar A , Dagar M, Herman J, Iacobuzio-Donahue C, Laheru D. CNS involvement in pancreatic adenocarcinoma: a report of eight cases from the johns hopkins hospital and review of literature. J Gastrointest Cancer. 2015;46(1):5-8. 10.1007/s12029-014-9667-y 25451139 PMC4451956

[30] Siegel RL , Giaquinto AN, Jemal A. Cancer statistics, 2024. CA Cancer J Clin. 2024;74(1):12-49. 10.3322/caac.21820 38230766

[31] Leidner R , Sanjuan Silva N, Huang H, et al. Neoantigen T-cell receptor gene therapy in pancreatic cancer. N Engl J Med. 2022;386(22):2112-2119. 10.1056/NEJMoa2119662 35648703 PMC9531755

[32] Qi C , Gong J, Li J, et al. Claudin18.2-specific car t cells in gastrointestinal cancers: phase 1 trial interim results. Nat Med. 2022;28(6):1189-1198. 10.1038/s41591-022-01800-8 35534566 PMC9205778

[33] Frank A , Alexis M, Katya M, et al. Brain metastasis from pancreatic cancer: our experience and systematic review. World Neurosurgery. 2022;166(10):e590-e598. 35863644 10.1016/j.wneu.2022.07.060

[34] Oweira H , Petrausch U, Helbling D, et al. Prognostic value of site-specific metastases in pancreatic adenocarcinoma: a surveillance epidemiology and end results database analysis. World J Gastroenterol. 2017;23(10):1872-1880. 10.3748/wjg.v23.i10.1872 28348494 PMC5352929

[35] Lee YT , Tatter D. Carcinoma of the pancreas and periampullary structures. Pattern of metastasis at autopsy. Arch Pathol Lab Med. 1984;108(7):584-587. 6547324

[36] Liu KH , Hung CY, Hsueh SW, et al. Lung metastases in patients with stage iv pancreatic cancer: prevalence, risk factors, and survival impact. J Clin Med. 2019;8(9):1402. 31500146 10.3390/jcm8091402 PMC6780197

[37] Hoshino A , Costa-Silva B, Shen TL, et al. Tumour exosome integrins determine organotropic metastasis. Nature. 2015;527(7578):329-335. 10.1038/nature15756 26524530 PMC4788391

[38] Reichert M , Bakir B, Moreira L, et al. Regulation of epithelial plasticity determines metastatic organotropism in pancreatic cancer. Dev Cell. 2018;45(6):696-711.e8. 10.1016/j.devcel.2018.05.025 29920275 PMC6011231

[39] Sahin IH , Elias H, Chou JF, Capanu M, O'Reilly EM. Pancreatic adenocarcinoma: insights into patterns of recurrence and disease behavior. BMC Cancer. 2018;18(1):769. 10.1186/s12885-018-4679-9 30055578 PMC6064173

[40] Campbell BK , Gao Z, Corcoran NM, Stylli SS, Hovens CM. Molecular mechanisms driving the formation of brain metastases. Cancers (Basel). 2022;14(19):4963. 10.3390/cancers14194963 36230886 PMC9563727

[41] Tsai C , Nguyen B, Luthra A, et al. Outcomes and molecular features of brain metastasis in gastroesophageal adenocarcinoma. JAMA Network Open. 2022;5(8):e2228083. 10.1001/jamanetworkopen.2022.28083 36001319 PMC9403772

[42] Dodoo GN , De B, Lee SS, et al. Brain metastases from biliary tract cancer: case series and clinicogenomic analysis. Oncologist. 2023;28(4):327-332. 10.1093/oncolo/oyac273 36715178 PMC10078902

[43] Integrated genomic characterization of pancreatic ductal adenocarcinoma. Cancer Cell. 2017;32(2):185-203.e113. 28810144 10.1016/j.ccell.2017.07.007 PMC5964983

[44] Zhang X , Mao T, Zhang B, et al. Characterization of the genomic landscape in large-scale chinese patients with pancreatic cancer. EBioMedicine. 2022;77:103897. 10.1016/j.ebiom.2022.103897 35231699 PMC8886010

[45] Negrao MV , Spira AI, Heist RS, et al. Intracranial efficacy of adagrasib in patients from the krystal-1 trial with krasg12c-mutated non–small-cell lung cancer who have untreated CNS metastases. J Clin Oncol. 2023;41(28):4472-4477. 10.1200/jco.23.00046 37327468 PMC10553074

[46] Ramalingam S , Skoulidis F, Govindan R, et al. P52.03 efficacy of sotorasib in kras p.G12c-mutated nsclc with stable brain metastases: a post-hoc analysis of codebreak 100. J Thorac Oncol. 2021;16(10):S1123. 10.1016/j.jtho.2021.08.547

[47] Rachakonda PS , Bauer AS, Xie H, et al. Somatic mutations in exocrine pancreatic tumors: association with patient survival. PLoS One. 2013;8(4):e60870. 10.1371/journal.pone.0060870 23565280 PMC3614935

[48] Blackford A , Serrano OK, Wolfgang CL, et al. Smad4 gene mutations are associated with poor prognosis in pancreatic cancer. Clin Cancer Res. 2009;15(14):4674-4679. 10.1158/1078-0432.CCR-09-0227 19584151 PMC2819274

[49] Utsunomiya T , Funamizu N, Ozaki E, et al. A case of radical resection for brain metastases of pancreatic cancer after curative chemotherapy for para-aortic lymph node metastases. Surg Case Rep. 2022;8(1):108. 10.1186/s40792-022-01461-2 35666369 PMC9170865

[50] Spectrum of breast cancer metastasis in brca1 mutation carriers: highly increased incidence of brain metastases. Ann Oncol. 2005;16(11):1846-1847. 15972278 10.1093/annonc/mdi351

[51] Golan T , Kanji ZS, Epelbaum R, et al. Overall survival and clinical characteristics of pancreatic cancer in BRCA mutation carriers. Br J Cancer. 2014;111(6):1132-1138. 10.1038/bjc.2014.418 25072261 PMC4453851

[52] Bindra RS. Penetrating the brain tumor space with DNA damage response inhibitors. Neuro Oncol. 2020;22(12):1718-1720. 10.1093/neuonc/noaa228 33059370 PMC7746934

[53] O’Connor CA , Park JS, Kaley T, et al. Leptomeningeal disease in pancreas ductal adenocarcinoma: a manifestation of longevity. Pancreatology. 2021;21(3):599-605. 10.1016/j.pan.2021.02.003 33582005 PMC8611374

[54] LEMKE J , BARTH TFE, JUCHEMS M, et al. Long-term survival following resection of brain metastases from pancreatic cancer. Anticancer Res. 2011;31(12):4599-4603. 22199336

[55] Ng JY-S , How E, Schwindack C, Lam A. Metastatic pancreatic adenocarcinoma presenting as an occipital haemorrhage. BMJ Case Rep. 2018;2018(8):bcr2018224354. 10.1136/bcr-2018-224354 PMC6078283 30068577

